# Self-Healing of Biocompatible Superhydrophobic Coatings: The Interplay of the Size and Loading of Particles

**DOI:** 10.1021/acs.langmuir.2c02795

**Published:** 2023-02-22

**Authors:** Nusret Celik, Furkan Sahin, Sultan Suleyman Ozel, Gulay Sezer, Nail Gunaltay, Mahmut Ruzi, M. Serdar Onses

**Affiliations:** † ERNAM − Erciyes University Nanotechnology Application and Research Center, 38039 Kayseri, Turkey; ‡ Department of Materials Science and Engineering, Erciyes University, 38039 Kayseri, Turkey; § Department of Pharmacology, Faculty of Medicine, Erciyes University, 38039 Kayseri, Turkey; ∥ UNAM − National Nanotechnology Research Center, Institute of Materials Science and Nanotechnology, Bilkent University, 06800 Ankara, Turkey

## Abstract

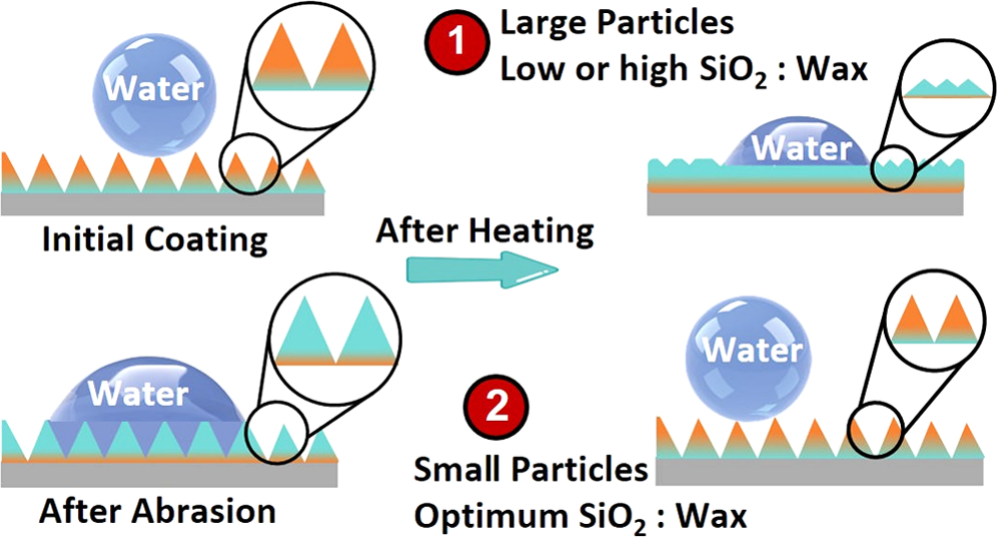

The broad application potential of superhydrophobic coatings is limited by the usage of environment-threatening materials and poor durability. The nature-inspired design and fabrication of self-healing coatings is a promising approach for addressing these issues. In this study, we report a fluorine-free and biocompatible superhydrophobic coating that can be thermally healed after abrasion. The coating is composed of silica nanoparticles and carnauba wax, and the self-healing is based on surface enrichment of wax in analogy to the wax secretion in plant leaves. The coating not only exhibits fast self-healing, just in 1 min under moderate heating, but also displays increased water repellency and thermal stability after healing. The rapid self-healing ability of the coating is attributed to the relatively low melting point of carnauba wax and its migration to the surface of the hydrophilic silica nanoparticles. The dependence of self-healing on the size and loading of particles provides insights into the process. Furthermore, the coating exhibits high levels of biocompatibility where the viability of fibroblast L929 cells was ∼90%. The presented approach and insights provide valuable guidelines in the design and fabrication of self-healing superhydrophobic coatings.

## Introduction

Superhydrophobic surfaces exhibit extreme repellency toward water, causing droplets to bead up and roll off easily. This behavior is due to the reduced contact area between a water droplet and the superhydrophobic surface where the droplet only contacts a small area of the solid around the apex of the rough surface and is supported by entrapped air pockets.^1^ Since the popularization of the term by the discovery of the “lotus effect” in 1997, various efforts have been made in fabricating superhydrophobic surfaces for technical applications such as self-cleaning, antifouling, anticorrosion, and anti-icing coatings.^2−7^ Almost all fabrication methods involved creating a rough surface in synergy with imparting low surface energy, therefore ensuring that water stays in the Cassie–Baxter state on the superhydrophobic surface.^8−10^ However, superhydrophobic coatings are susceptible to damage, upon which a liquid droplet impales the valleys of the rough surface via replacing air pockets, transitioning to the Wenzel state.^1^ Therefore, weak durability is one of the main drawbacks impeding the real-life application of superhydrophobic coatings.^11^ Furthermore, the usage of low surface energy imparting agents such as perfluorinated chemicals and petroleum-based long-chain hydrocarbons is another emerging issue.^12,13^ The concern is about health and the environmental impact of these chemicals: since they are very stable, they do not biodegrade and therefore persist in the environment, eventually ending up in plants and animals, causing various pathological responses that can lead to various health issues.

Over the last decade, various approaches have been developed to overcome these two issues. The durability of superhydrophobic coatings can be improved using microscopic protective structures prepared by lithography and molding methods.^14,15^ Another approach is making the bulk structure superhydrophobic via 3D printing technology or solution-based molding processes.^16−18^ Environmental concerns over the usage of problematic chemicals can be mitigated using ecofriendly natural waxes such as carnauba wax and beeswax.^13,19−22^ However, artificial superhydrophobic coatings prepared from these natural waxes generally lack robustness, in contrast to the natural superhydrophobic surfaces of a lotus leaf.^23,24^ Research revealed that the robustness of natural superhydrophobic surfaces is due to a two-tier hierarchical rough surface structure, and most importantly, the self-healing ability to regenerate both roughness and low surface energy after degradation.^25,26^ This self-healing property inspired many researchers to design superhydrophobic surfaces using natural materials with the self-healing ability to compensate for the weak durability issues of many artificial superhydrophobic surfaces.^20,21^ Wang et al. fabricated a self-healing superhydrophobic surface by replicating the surface of a lotus leaf using poly(dimethylsiloxane) (PDMS), followed by depositing mixtures of PDMS and *n*-nonadecane.^27^ After degradation via exposure to oxygen plasma, the coating is able to recover superhydrophobicity spontaneously within 20 min. Li et al. fabricated a near-IR light-responsive self-healing superhydrophobic coating using polyurethane and polydopamine-modified ZnO NPs.^28^ The scratch-degraded coating can regain superhydrophobicity after either heating at 70 °C for 1 h or 30 s exposure to near-IR laser light. A more ecofriendly approach was demonstrated recently by Sun et al. where natural diatom and beeswax were used to prepare a self-healing superhydrophobic coating.^29^ The superhydrophobicity of the coating was damaged by water jet impact, and self-healing was achieved via heating at 80 °C for 20 min.

An ecofriendly superhydrophobic coating that is self-healing or healable under moderate conditions is thus needed. Moreover, understanding the mechanism of self-healing needs to be vastly improved for the effective design of self-healing superhydrophobic surfaces. Superhydrophobic coatings composed of silica nanoparticles and wax materials have been previously studied.^30−33^ Most of these studies involved chemical modification of the silica nanoparticles to impart hydrophobicity. Only a few reports based on silica nanoparticles and wax have shown heating-induced self-healing behavior.^29^ However, the effect of size and loading of particles and the underlying mechanism were not studied in detail. In this work, we present the preparation of self-healing superhydrophobic coatings using hydrophilic silica NPs and plant-based carnauba wax and explore the impact of various parameters on the self-healing ability of the superhydrophobic coating. This coating can quickly and repeatedly recover its excellent water repellency through simple heat treatment (e.g., 90 °C for 1 min) after being damaged by abrasion or peeling. Furthermore, the wetting properties, composition, and morphology of surfaces after the damage and healing processes are studied. The parameter window for obtaining self-healing superhydrophobic coatings is obtained as a function of the size and loading of the silica particles.

## Materials and Methods

### Materials

Chloroform (99%), toluene (99%), carnauba wax, beeswax, and spherical hydrophilic SiO_2_ nanoparticles of different sizes (11 nm, 90 nm, 260 nm, 4 μm, 8 μm, 20 μm) were purchased from Sigma-Aldrich. Ethanol (99%), paraffin wax, and acetone (99%) were obtained from Merck. These chemicals were used as received. Glass slides (2.6 cm × 7.6 cm) were bought from Isolab Inc. The L929 (murine fibroblast) cell line was purchased from the American Type Culture Collection (ATCC, Manassas, VA), cultured in Dulbecco’s modified Eagle’s medium (DMEM, Sigma Chemical Company, St. Louis) supplemented with 10% fetal bovine serum (Gibco BRL), 1% penicillin/streptomycin (Sigma-Aldrich, Germany), and 1% glutamine (Gibco, U.K.) at 37 °C in a humidified atmosphere of 5% CO_2_.

### Preparation of the Coating

The superhydrophobic coating was prepared following similar procedures as described in our previous work.^30^ Specifically, 0.4 g of carnauba wax was added to 20 mL of chloroform in a test tube and heated at 90 °C under magnetic stirring until complete dissolution. Afterward, 0.2 g of unmodified SiO_2_ nanoparticles was added, while the stirring was continued for 20 min. In the end, a clear and stable (>1 month) dispersion was obtained (Figure S1). Unless otherwise stated, the diameter of the nanoparticles was 11 nm. Finally, the prepared colloidal suspension was spray-coated onto glass substrates (1 × 1 cm^2^) held at 45° from 20 cm using an airbrush with a nozzle diameter of 0.35 mm at a pressure of 4.0–4.5 bar. The substrate was subjected to 10 cycles of spray-coating and left to dry at room temperature for 1 h. The procedures of preparing superhydrophobic coatings using hydrophilic silica NPs of various sizes and various waxes (carnauba wax, beeswax, paraffin wax) and different solvents (toluene, chloroform) are the same.

### Characterization

To determine the surface wettability, the contact angle (CA) and the sliding angle (SA) were measured with an optical tensiometer (Attension, Theta Lite). CA and SA were measured at three different locations using water droplets of 5 and 10 μL, respectively. The reported results are arithmetic averages obtained from these three measurements. The surface morphology of samples was imaged via a scanning electron microscope (SEM) (Zeiss EVO LS10) at 25 kV. Surface topography was examined with a profilometer (Bruker-DektakXT). Before measurements, the sample surface was coated with a thin layer of gold via sputtering. The chemical composition was characterized via FTIR using the ATR mode (LUMOS II, Bruker) and X-ray photoelectron spectroscopy (XPS). For the XPS measurements, a Thermo Scientific K-Alpha spectrometer with a monochromatic Al Kα source (1486.7 eV) was used. The XPS data were calibrated against adventitious C 1s. The thermal property of the materials was investigated via differential scanning calorimetry (DSC, METTLER1). Specifically, 4–7 mg of the sample was placed in an aluminum pan and heated to 200 °C, equilibrated for 5 min, and then cooled to room temperature. The heating and cooling rate was 10 °C/min.

### Mechanical Durability Tests

The mechanical durability of the coatings was investigated using a linear abrasion test and a tape peeling test. In the linear abrasion test, the coated sample was glued under 200 g (19.6 kPa) of load and moved on an aluminum foil while the coated side was touching the aluminum foil. A movement distance of 10 cm was counted as one cycle. In the tape peeling test, an adhesive tape was glued to the coated surface of the sample and kept under 200 g of load for 1 min, and then the tape was removed from the surface.

### Degradation and Thermal Healing

Degradation of the superhydrophobic coating was performed by abrading the sample against an aluminum foil under a load of 19.6 kPa. For healing, the degraded samples were first cleaned by blowing dry N_2_, followed by placing them on a preheated (90 °C) hotplate for one min. The characterization was conducted after allowing the retrieved sample to cool to room temperature.

### Cytotoxicity Test

Cytotoxicity was evaluated according to the ISO10993-5-2009 regulation, where the L929 cell line was chosen, and an MTT cell viability assay was performed. To obtain extraction solutions, samples were cut into square pieces (2 × 2 cm^2^) and sterilized by UV irradiation for 40 min. Then, samples were placed in six-well tissue culture plates and filled with a culture medium (DMEM) with an extraction ratio of 4 cm^2^/0.666 mL at 37 °C in a 5% CO_2_ incubator for 24 h. L929 cells were seeded into 96-well culture plates at a density of 6 × 10^3^ cells/well in 100 μL of a culture medium and incubated for 24 h at 37 °C in a humidified atmosphere containing 5% CO_2_ in air. After this time, culture media were replaced with 100 μL of the sample and control group extracts (the extraction medium without the sample and only a glass slide were used as control groups). Cells were examined microscopically after 24 h of incubation to assess the general morphology of cells. Then, the cells were incubated with 10 μL of a 3-(4,5-dimethylthiazol-2-yl)-2,5-diphenyltetrazolium bromide solution (MTT, 5 mg/mL, Sigma-Aldrich, Germany) for 3 h. Formazan crystals were dissolved in 100 μL of dimethyl sulfoxide, and the absorbance (OD) was measured with a microplate reader (Promega Multireader Glomax) at 560 nm. Cell viability was calculated by the following equation
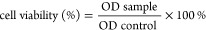


Experiments were performed in triplicate, and mean OD values were normalized to the control group and represented as cell viability (%).

### Statistical Analysis

The statistical significance among multiple groups was assessed using one-way analysis of variance (ANOVA) followed by the Tamhane *T*^2^ post-hoc test. Significance was accepted at a *p*-value of less than 0.05 using SPSS 21.0 (IBM). The results are expressed as mean ± standard deviation (SD) of three independent assays.

## Results and Discussion

**Figure 1:**
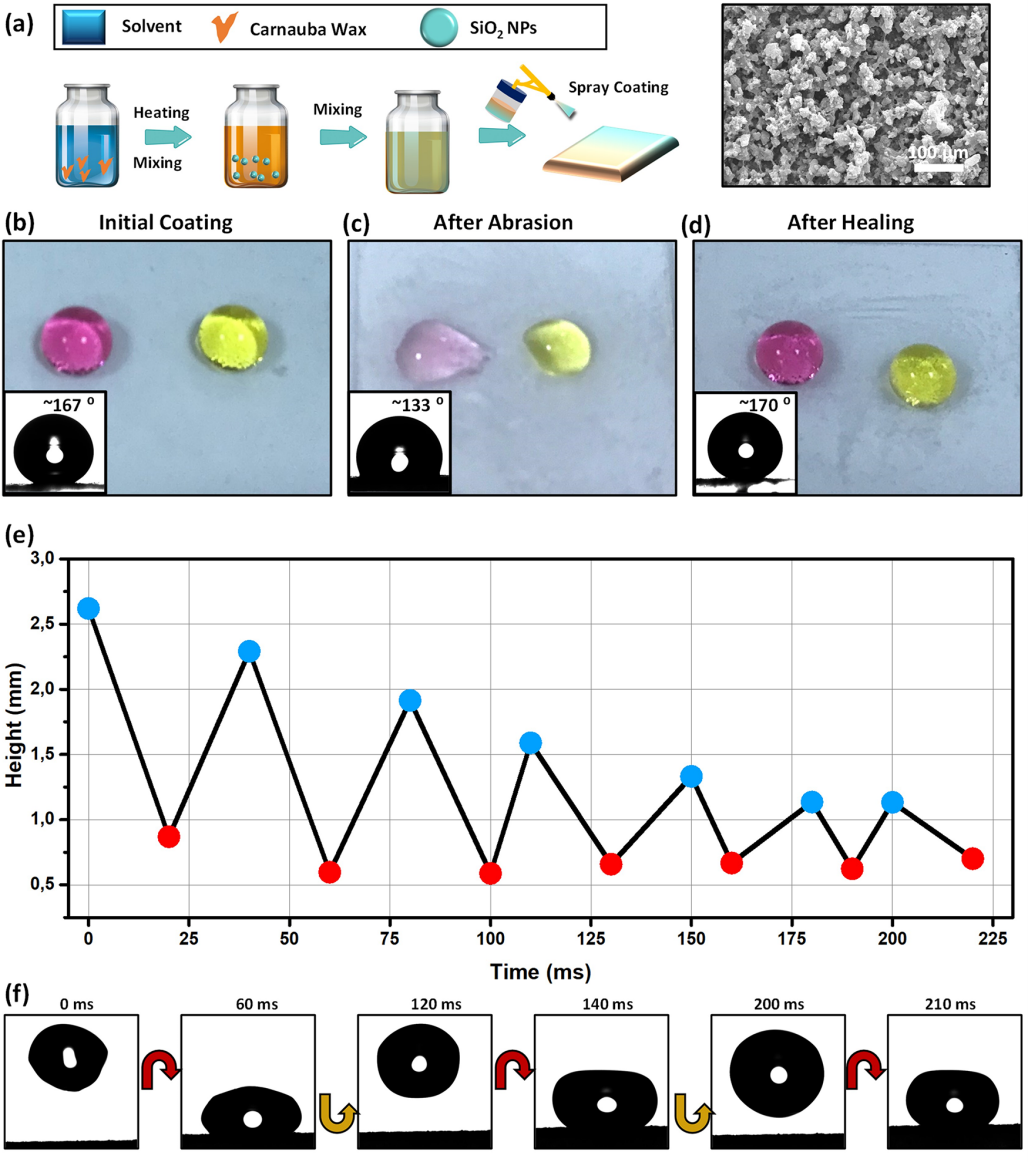
Preparation of self-healing superhydrophobic coatings. (a) Illustration of the preparation and deposition of the superhydrophobic coating. Also shown is a SEM image of the coating. (b–d) Photographs of dyed water droplets on the superhydrophobic coating (b) before abrasion, (c) after abrasion, and (d) after healing (at 90 °C for 1 min). The insets at the lower left corner are the images of a 10 μL water droplet on the corresponding surfaces. (e) Temporal evolution of the center of gravity of a ∼6 μL water droplet (*R*_0_ = 1.2 mm) after release from a height of ∼10 mm (tip to surface) and bouncing on the healed superhydrophobic surface with an initial impact speed of 0.16 m/s. (f) Snapshots of the bouncing droplets.

Figure 1a illustrates the main steps of fabricating the superhydrophobic coating, which is prepared by dissolving carnauba wax in hot chloroform to get a homogeneous dispersion, followed by adding hydrophilic SiO_2_ NPs. The final dispersion is coated on the substrate and left to dry at room temperature. Spray-coating of wax/hydrophilic SiO_2_ NPs leads to a textured surface (see the SEM image), which exhibits superhydrophobic behavior with a water CA of 167 ± 2° and an SA of 4 ± 1°. This is interesting since the majority of superhydrophobic coatings reported in the literature are obtained using nanoparticles modified with hydrophobic agents such as alkylsilanes.^8,10^ Therefore, the key advantage of our approach is the direct fabrication of superhydrophobic coatings using commercial hydrophilic NPs without the need for chemical modification. However, superhydrophobic coatings prepared directly from unmodified NPs are expected to be fragile, since wear can reveal the hydrophilic nanoparticles, thereby reducing the water repellency.^34^ This behavior was observed as illustrated in Figure 1b–d. After abrasion, the water CA decreases to 133 ± 2°. Nonetheless, the superhydrophobicity can be recovered (CA: 170 ± 2°, SA: 1°) by heating (at 90 °C for 1 min) the degraded sample to above the melting point (∼83 °C) of carnauba wax (Figure S2), probably due to migration of wax molecules to the air/solid interface.^35^ Healing is also possible at low temperatures (60 °C) but requires a long time (∼18 h). Besides the value of the water CA and SA, multiple bouncing of an impinging droplet on a surface is another characteristic of a superhydrophobic surface.^36−38^ While a water droplet (6 μL) rebounds only two times on the initial coating (Figure S3 and Video S1), it rebounds five times on the healed surface under the same condition (Figure 1e). The increased rebound after healing is another indication of the improvement of the superhydrophobicity.^37^ It should be noted that besides chloroform, toluene can also be used as a solvent, even though the dispersion is unstable (Figures S4 and S5). Another natural material, beeswax, can also be used to prepare self-healing superhydrophobic coatings instead of carnauba wax (Figure S6).

**Figure 2:**
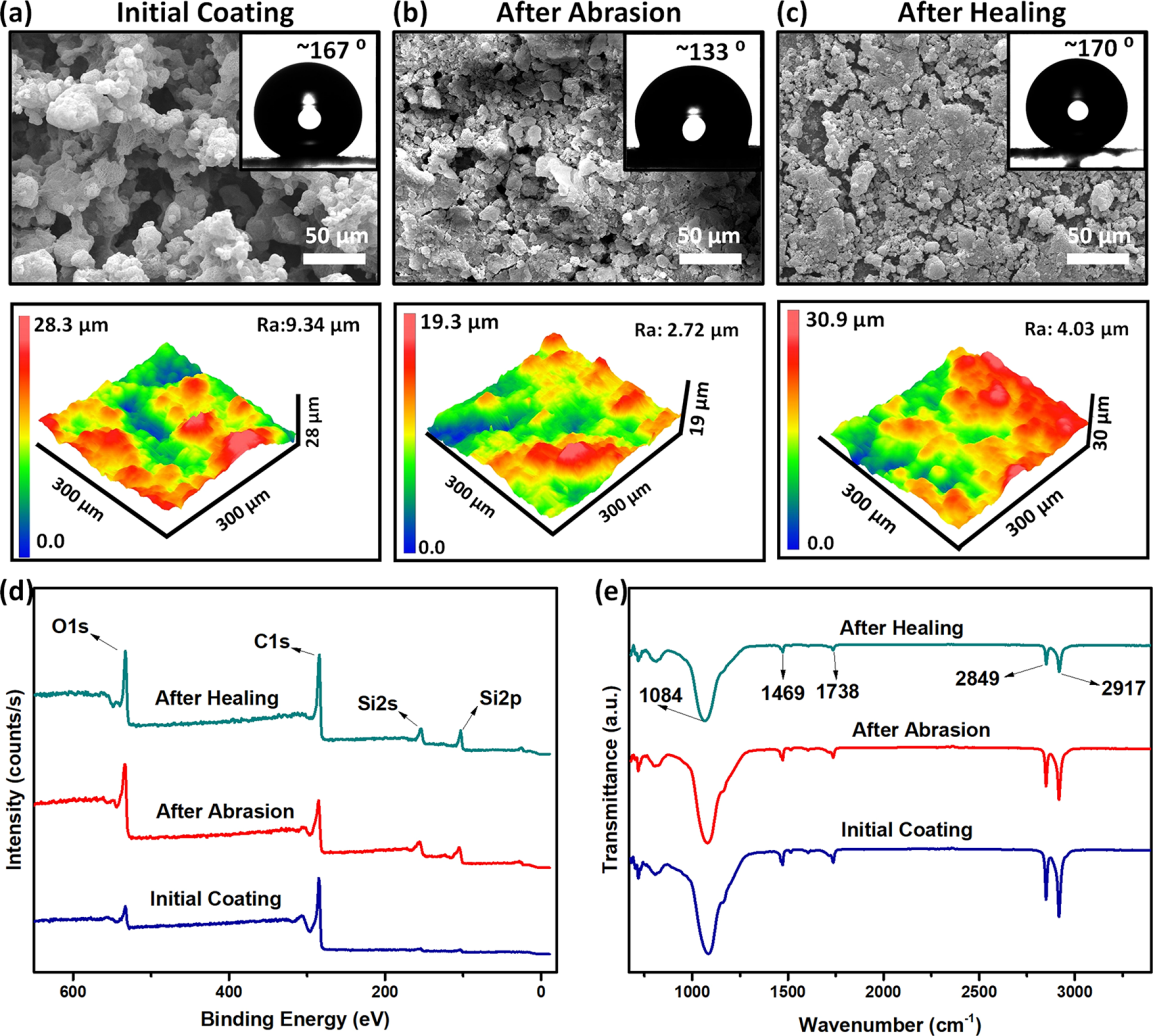
Surface characterization of the self-healing superhydrophobic coating. (a–c) SEM (top row) and 3-D height profile images (bottom row) showing the morphology and topography of the surfaces (a) initial coating, (b) abrasion, and (c) heating. (d) XPS survey spectra and (e) FTIR spectra of the surfaces after each process.

The large surface roughness and low surface energy are two conditions that are necessary for achieving superhydrophobicity.^26^ Therefore, we investigated the change in surface morphology, topography, and as well as surface chemistry of samples during the self-healing process. As shown in Figure 2a,b, the surface morphology and topography of the sample changed significantly after the abrasion. The initial superhydrophobic coating with a rough (*R*_a_ = 9.34 μm) surface becomes smoother (*R*_a_ = 2.72 μm) after the abrasion and loses superhydrophobicity (CA ∼133°). However, after the heating, the surface regains some roughness (*R*_a_ = 4.03 μm) and becomes superhydrophobic (CA: 170 ± 2°, SA: 1°) again. It is interesting that even though the surface does not fully regain roughness after healing, the superhydrophobicity improved. It is well-known from Cassie–Baxter and Wenzel equations that the roughness and CA are correlated. In the presented system, the coating is composed of two components with significantly different surface energies. As a result, the variation in the surface composition is coupled with the change in roughness. In addition to the surface morphology and topography, the chemical composition of the surface also changes noticeably upon abrasion and heating. As shown in the XPS spectra in Figure 2d, there is a noticeable change in the composition. The initial coating contains mostly C atoms (83.61%) and only a low amount of Si atoms (4.65%) (Table S1). After abrasion, C atomic content decreased to 53.21%, while Si amount increased to 17.06%, which is of SiO_2_ NPs’ origin, confirming the exposure of the hydrophilic silica NPs to the air interface. After the abraded sample is heated, the C atomic content increased to 63.22%, while the Si atomic percentage slightly decreased to 14.73%. Furthermore, the FTIR spectra (Figure 2e) of the surfaces during the degradation–healing process do not show a noticeable change, and consist of characteristic peaks of silica NPs (1084 cm^–1^, Si–O stretch) and carnauba wax (C–H stretch at 2917 cm^–1^ and 2849 cm^–1^; C=O stretch at 1738 cm^–1^; CH_3_ bending at 1469 cm^–1^).^39^ These results indicate that there is no chemical change that occurred during the process. It should be noted that XPS only probes the surface layer of a few nm, while the FTIR (in ATR mode) probes down to a few μm. Therefore, the intensity profile of the XPS data is an indication of the true surface composition. Therefore, the chemical analysis of the surfaces shows that upon abrasion, the hydrophilic silica NPs are exposed to the surface, leading to the degradation of hydrophobicity, and heating the surface enables the migration of hydrophobic carnauba wax molecules to the surface, leading to the restoration of superhydrophobicity.

**Figure 3:**
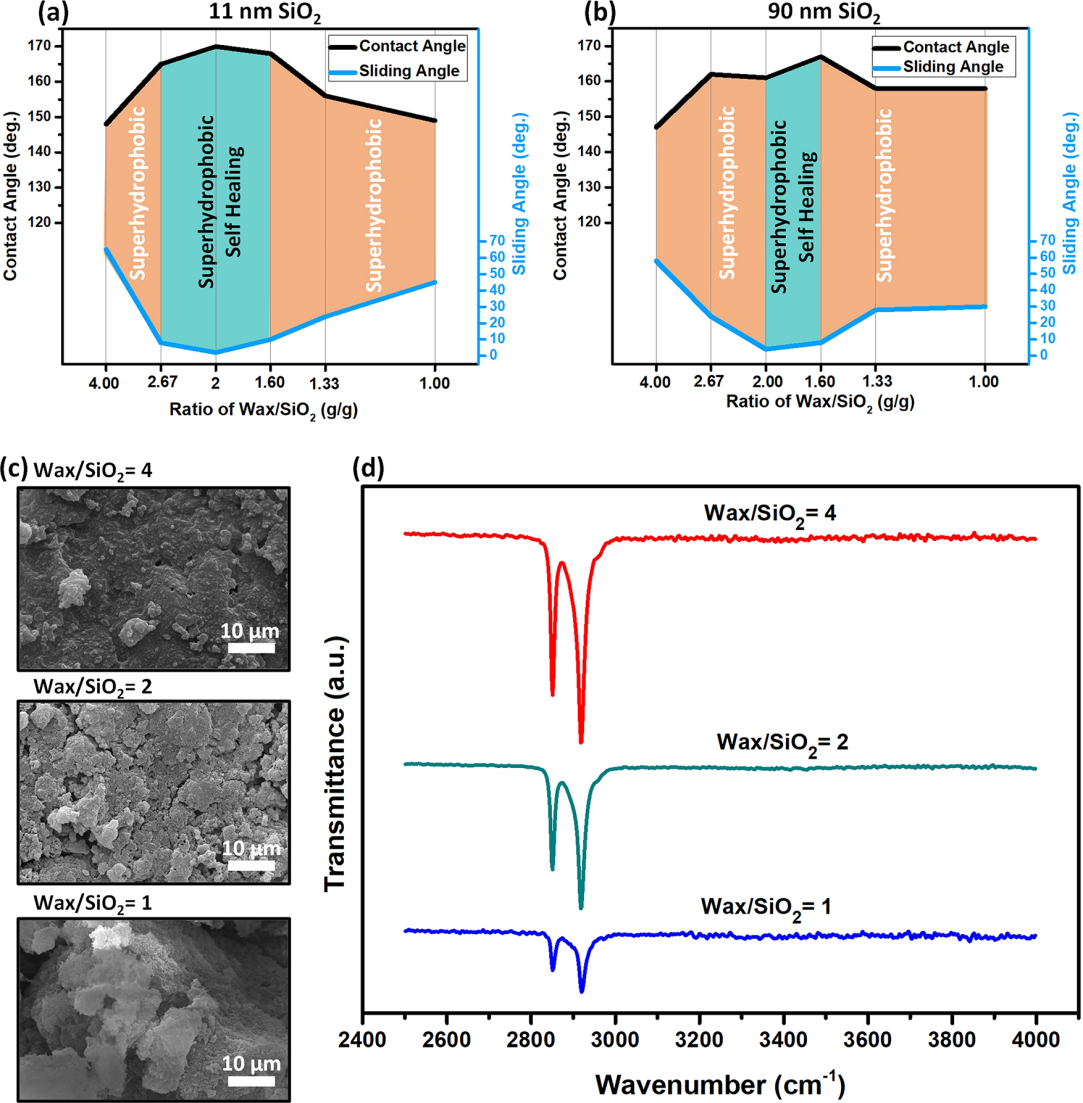
Impact of the mass ratio of SiO_2_ NPs to the solvent on the superhydrophobicity and self-healing ability. The effect of the mass ratio of SiO_2_ NPs for (a) particle size of 11 nm and (b) particle size of 90 nm. (c) SEM images and (d) FTIR spectra of the coatings prepared from wax/SiO_2_ NP ratios of 4, 2, and 1 while the solvent volume is kept at 20 mL. The images and spectra are taken after healing (heating the abraded samples).

The effect of the number of NPs and hydrophobic molecules used to prepare superhydrophobic coatings on the wetting properties is well-documented.^19,39−41^ To examine this effect, we prepared superhydrophobic coatings using 0.4 g of carnauba wax and 0.1–0.4 g of hydrophilic SiO_2_ NPs. Figure 3 presents the wetting properties as a function of the weight ratio of SiO_2_ NPs to carnauba wax. The surface exhibits superhydrophobic behavior in a relatively large window. However, the self-healing behavior is not observed in all coating compositions that display superhydrophobicity. When the size of silica NPs is 11 nm, the coating exhibits self-healing properties only when the ratio of SiO_2_ NPs to wax is between 2.67 and 1.60 (Figure 3a). For larger silica NPs (90 nm), the range becomes even narrower (2.00–1.60) (Figure 3b). Both the surface roughness and surface energy could be the primary reason for the observed self-healing ability dependence on the SiO_2_ NP loading. To uncover the mechanism, the surface morphology, and composition of the coatings prepared using wax/SiO_2_ NPs of 4 (lowest), 2 (optimal), and 1 (largest) were characterized after the heating. As can be seen from the SEM images (Figure 3c), when SiO_2_ content is low, the surface roughness is low and the amount of carnauba wax on the surface is large, as inferred from the intensity of the CH stretch peaks of carnauba wax at 2850 and 2920 cm^–1^ (Figure 3d). This result is consistent with the observation that when only wax is used, it forms a rather smooth thin film upon heating (Figure S7). On the other hand, when the SiO_2_ NP ratio is increased to 1, the amount of carnauba wax molecules on the surface is low (inferred from FTIR spectra) while the roughness is large. Only when wax/SiO_2_ NP = 2 is used, the surface exhibits large roughness and carnauba wax content. Thus, a synergic combination of surface roughness and low surface energy is necessary for obtaining a self-healing superhydrophobic surface using hydrophilic silica NPs and carnauba wax. Nonetheless, this empirical observation does not shed light on the microscopic mechanism, which needs to be elucidated. Upon melting, wax molecules, and to a limited extent silica NPs, can diffuse around and interact with other wax molecules or NPs. Therefore, there is a competition between the interaction of the wax molecules and silica NPs in a melt, leading to clusters of wax and wax-covered silica NPs upon cooling. Accordingly, the ratio of the wax to the silica NP is higher (1.6) for self-healing and superhydrophobic coatings than the ratio (1.0) for just superhydrophobic coatings. On the other hand, when the amount of wax molecules is in excess, the silica NP is covered by thick layers of wax. Even though the initial coating is still superhydrophobic, the extra wax molecules melt upon heating, filling in gaps between NPs, resulting in reduced roughness, hence, reduced CA and increased SA. Since the surface area of smaller NPs is larger, small silica NPs can accommodate more wax (2.67) than larger NPs (2.0) to retain the self-healing property.

**Figure 4:**
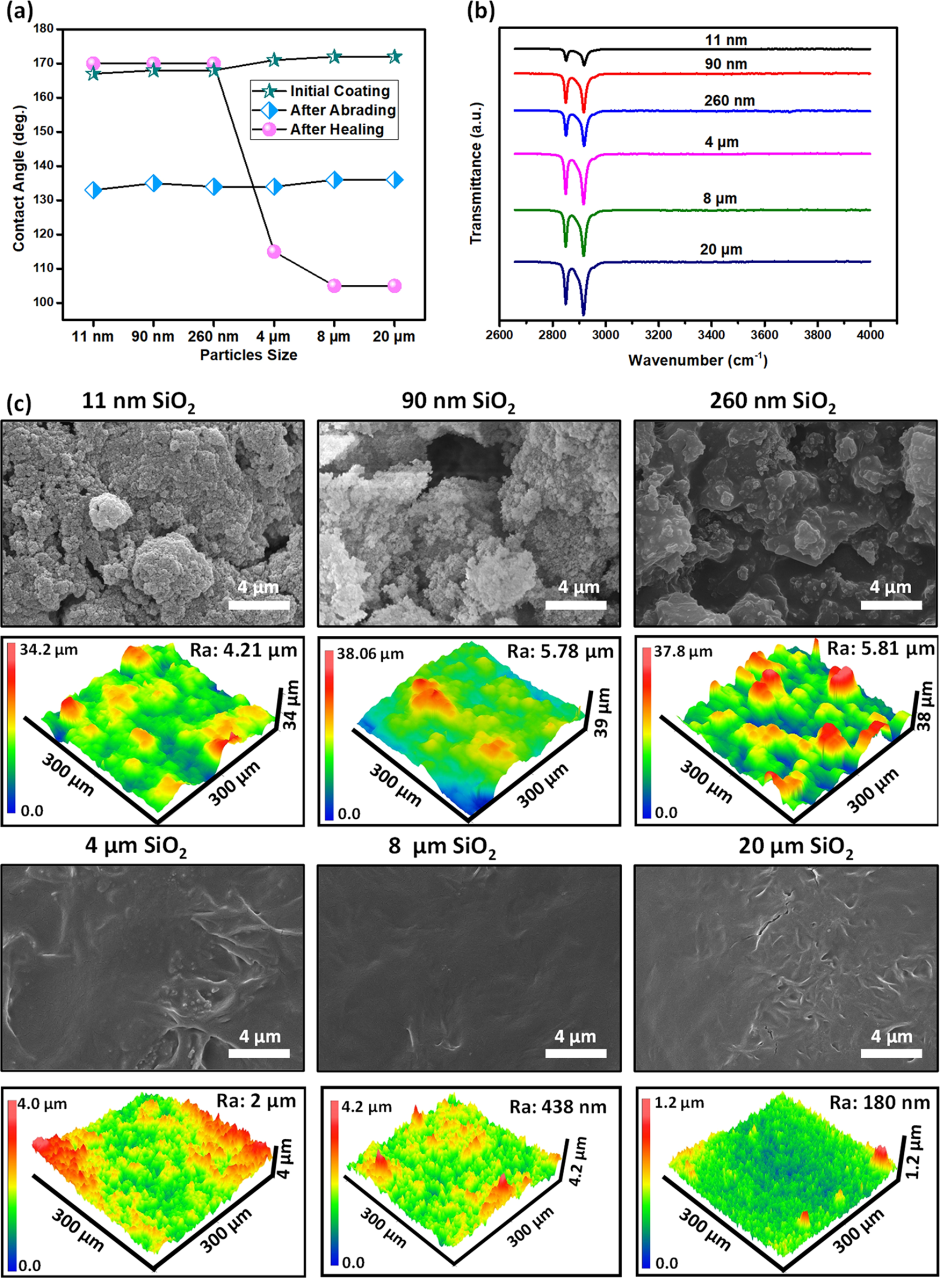
Effect of particle size on superhydrophobicity and self-healing property. (a) Water CA on the coatings prepared from silica NPs of different sizes before and after abrasion, as well as after healing. (b) FTIR spectra of coatings prepared from silica NPs of different sizes after healing, showing the C–H stretch region of carnauba wax molecules. (c) Surface morphology and topography of the coatings prepared from silica NPs of various sizes after healing.

The results discussed in the previous section indicate that the self-healing ability of the superhydrophobic coating prepared from 0.4 g of carnauba wax and 0.2 g of hydrophilic silica NPs exhibits some dependence on the size of silica NPs. Therefore, the dependence of hydrophobicity and self-healing ability on the size of particles is further examined for silica NPs of various sizes from 11 nm up to 20 μm (Figure 4; also Table S2 for SA). When the particle size increases, the number of SiO_2_ particles per unit mass decreases, so it is expected that there is an upper limit on the size of the particle above which the superhydrophobic coating does not exhibit self-healing properties. As shown in Figure 4a, for particle sizes up to 260 nm, the surface shows both superhydrophobic and self-healing properties. For particles larger than 260 nm, the surface exhibits superhydrophobicity but not self-healing properties. Further chemical and structural characterization are performed on these coatings after healing. As shown in the FTIR spectra (Figure 4b), the amount of carnauba wax molecules on the surface increases as the particle size increases. This observation implies the increased accretion of wax molecules onto the surface for coatings prepared from large silica NPs since the same amount of material was used. Therefore, the size and surface area of the particles play a key role in the self-healing ability. As shown in Figure 4c, when the size of the particle increases up to 260 nm, the surface roughness increases slightly. However, for the coatings prepared from silica NPs whose sizes are larger than 260 nm, the roughness, on the other hand, starts to decrease. For example, the surface roughness of the coating prepared from silica NPs of 11 nm is 4.21 μm, while the roughness is only 180 nm when the size of silica particles is 20 μm. The observed trend of decrease of water CA with the surface roughness is consistent with the trend predicted by the Cassie–Baxter equation and previous studies.^40,42^ As can be seen from the FTIR spectra and imaging (SEM and profilometry), for larger particles (>260 nm), a continuous film of carnauba wax forms on the surface of the coating after heating, resulting in reduced roughness.

**Figure 5:**
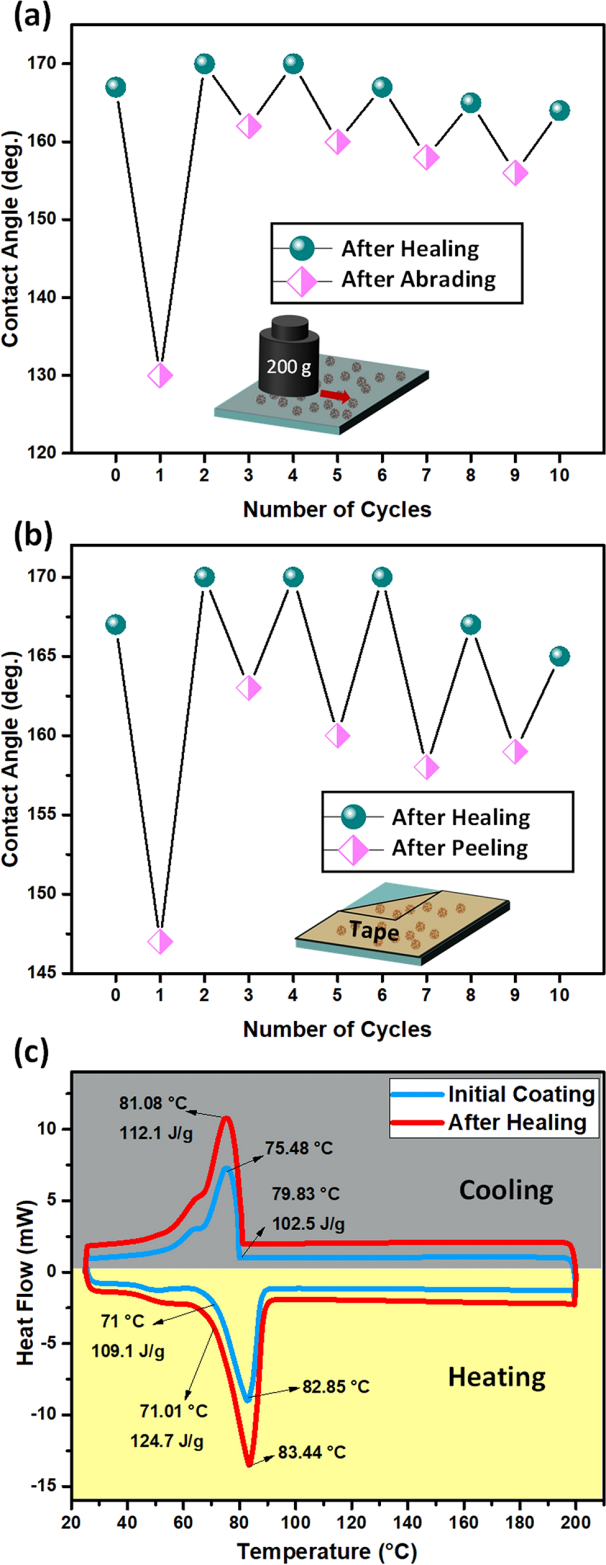
Evaluation of the durability of the self-healing superhydrophobic coating. The change of water CA of the coating as a function of (a) abrasion/healing and (b) tape peeling/healing cycles. The inset in each figure is an illustration of the corresponding tests. The corresponding SA values are provided in Table S3. (c) DSC curve of the initial (blue) superhydrophobic coating and after the first cycle (red) of abrasion/healing.

One advantage of a self-healing superhydrophobic surface is the possibility of regaining superhydrophobicity after degradation.^26^ To evaluate the self-healing ability, the superhydrophobic coating is subjected to cycles of degradation–healing via linear abrasion and tape peeling tests, followed by heating. As shown in Figure 5a,b, the original coating loses superhydrophobicity after the first cycle of both linear abrasion and tape peeling, where the water CA decreased by 47 and 20°, respectively. In addition, the SA increased significantly when a water droplet of 6 μL stuck to the degraded surface without sliding (Table S3). However, the coating regains superhydrophobicity after heating at 90 °C for only 1 min, after which the water CA increased to 170° and SA decreased to 2°. Interestingly, further abrasion and tape peeling after the first cycle of degradation/healing do not degrade superhydrophobicity, while the CA value is decreased only by ∼10° and SA values increased to 14° at most (Table S3). This result indicates that the stability of the coating is increased significantly after the first degradation/heating cycles. To further evaluate the stability of the coating, thermal analysis is performed. Figure 5c shows the DSC curve of the superhydrophobic coating before and after the first cycle of degradation/healing. The initial coating starts to melt at 71 °C (blue curve) and displays a melting temperature of 82.85 °C, while the enthalpy of melting is 109.1 J/g. However, after the first cycle of damage/healing, the melting temperature increases slightly (0.59 °C), while the enthalpy of melting reaches 127.4 J/g, a ∼17% increase. Similarly, the enthalpy of solidification (by 8.5%), as well as the solidification temperature (by 7.4%), increases noticeably after the first cycle of abrasion/healing. The noticeable increase of enthalpy after degradation/heating is consistent with the increased durability against linear abrasion and tape peeling. It should be noted that pure carnauba wax melts and forms a homogeneous thin film (Figure S7) upon heating at 90 °C. Therefore, the presence of silica NPs seems to increase the thermal stability of carnauba wax where the composite coating is stable up to 200 °C. The increased stability is significant since generally fluorinated polymers are used to encapsulate waxes to increase their thermal stability.^31^ The increase of enthalpy after healing is interesting in itself and is probably due to the relaxation of the strained configuration of wax molecules and/or the formation of hydrogen bonding with surface silanol groups (Si–OH) of the silica NP surface. The latter interaction is possible because carnauba wax was composed mostly of long-chain esters,^43^ where the carbonyl group (C=O) can act as a hydrogen bond acceptor, while the silanol group donates one. It should be noted that the carnauba wax molecules associate with each other in a head-to-tail fashion via dispersion forces, which is generally weaker than hydrogen bonding.^20^ Therefore, both strain relaxation and the formation of hydrogen bonds can lead to increased stability.

**Figure 6:**
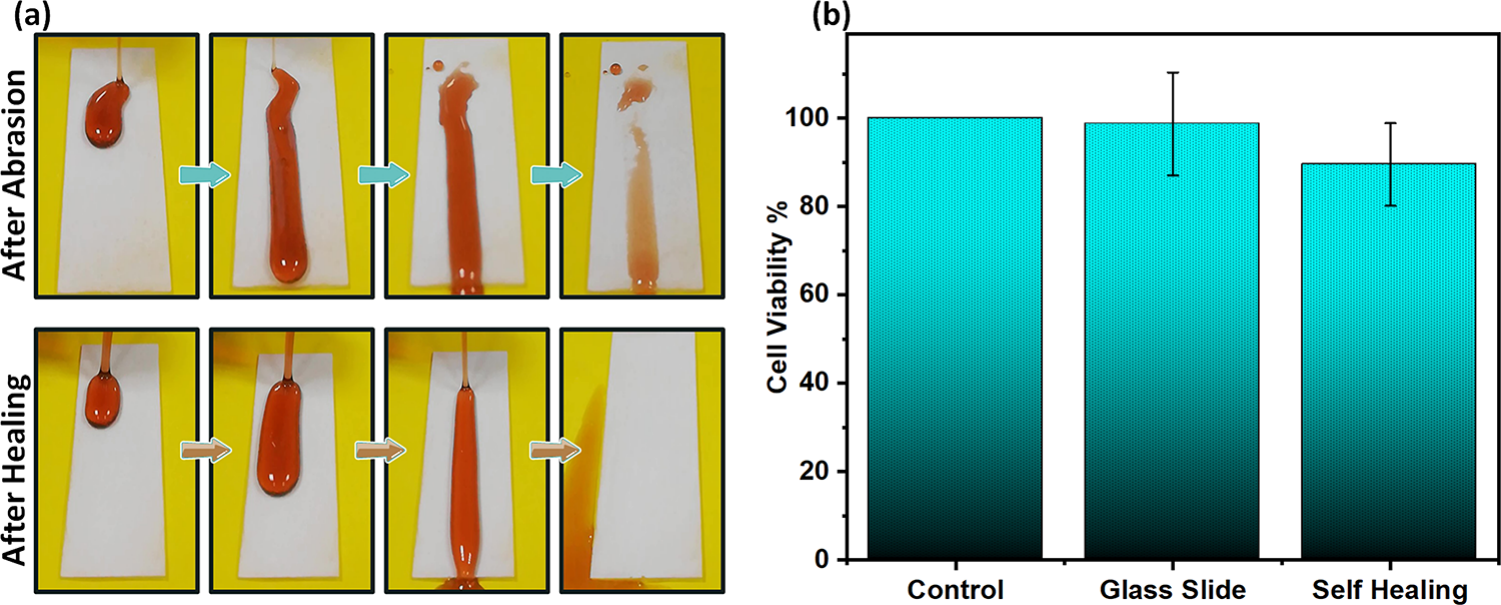
Demonstration of the application of the biocompatible self-healing superhydrophobic coating. (a) Self-cleaning against common liquid food such as pomegranate syrup. Top: damaged surface. Bottom: healed superhydrophobic surface. (b) Effects of the sample and control extracts on the viability of L929 cells at 24 h of exposure. Data express the percentage of cell viability obtained by the MTT test (*n* = 3).

The degradation/healing tests clearly demonstrate the self-healing ability of the superhydrophobic coating. It should be noted here that almost all previous reports of self-healing superhydrophobic coatings used air plasma to demonstrate degradation.^26^ Therefore, it is not clear how these self-healing superhydrophobic coatings perform under realistic degradation conditions, such as abrasion and peeling, as conducted in this work. Furthermore, for practical applications, the self-healing superhydrophobic coating should be easily applicable to various types of surfaces such as metals, cardboards, and plastics. The self-healing superhydrophobic coating reported in this study can be easily applied to different substrates and show self-healing ability without damaging the substrates upon mild heating for a short time. Importantly, the healing process should be spontaneous or only involve mild input that can be applied on a large scale. For this, an ITO plate was coated with the superhydrophobic coating, and healing was achieved after only 3 min of applying moderate voltage (see Figure S8 and Video S2). Another potential application of self-healing superhydrophobic coatings is for food packaging to reduce food waste.^44,45^ Therefore, we evaluated the antifouling ability of the superhydrophobic coating against common liquid food such as pomegranate syrup, as shown in Figure 6a. Here, some samples stick to the surface after degradation. However, after healing via heating, no sample is visible on the superhydrophobic surface, displaying self-cleaning ability. So far, glass has been used as a substrate. But apart from glass, paper and nylon were also used as a substrate. The coatings prepared using these substrates also show self-healing properties (Figure S9). Besides, the self-healing superhydrophobic surface shows great biocompatibility, as analyzed with the fibroblast L929 cell line.^46,47^ The mean viability values of sample and glass slide extracts treated cells were 89.53% and 98.72%, respectively (Figure 6b). Our results showed that the extract of the sample did not cause a significant change in the viability of L929 cells (*p* > 0.05). According to ISO 10993-2009, a sample is considered noncytotoxic if the percentage vitality value is >70%.^48,49^ The morphology of the sample extract and only medium-treated cells was also similar (Figure S10).

## Conclusions

In this work, a self-healing superhydrophobic coating was prepared directly using low-cost and industrially available hydrophilic silica nanoparticles and plant-based carnauba wax. Here, no chemical modification of the silica nanoparticles is needed. A self-healing strategy was proposed to improve the durability of the superhydrophobic coating. Self-healing tests performed under realistic conditions reveal that damaged coating regains superhydrophobicity after heating at 90 °C for only 1 min. The detailed investigation reveals that a self-healing superhydrophobic coating can be obtained using hydrophilic SiO_2_ particles up to 260 nm in size and moderate loading. Either too high or too low particle loading inhibits the self-healing ability due to either the low surface roughness or insufficient hydrophobicity. In addition, since the self-healing superhydrophobic surface shows biocompatibility when analyzed with the fibroblast L929 cell line, this coating can be used in diverse applications including food packaging and biomedical devices. This work has established a relationship among key parameters for the fabrication of self-healing superhydrophobic coatings through direct usage of unmodified silica nanoparticles and natural wax materials. Improving the practical applicability of the presented approach motivates the exploration of environment-friendly solvents to replace chloroform.

## Supporting Information Available

Surface composition inferred from XPS, sliding angle of the coatings, droplet bouncing, additional SEM and profilometer images, demonstration of healing of the large area surface via electrical heating, L929 cells’ optical images, and demonstration of healing on different substrates (PDF)
Bouncing of a 10 μL water droplet (Video S1) (MP4)
Achieving self-healing via electrical heating (Video S2) (MP4)
