# Supplementary material for: Self-Healing of Biocompatible Superhydrophobic Coatings: The Interplay of the Size and Loading of Particles
Source: Langmuir. 2023 Feb 22;39(9):3194–203. doi: 10.1021/acs.langmuir.2c02795 (PMC9996814; doi:10.1021/acs.langmuir.2c02795)
Supplement: Supplementary file 1 — la2c02795_si_001.pdf [file la2c02795_si_001.pdf]

Supporting Information

Self-Healing of Biocompatible Superhydrophobic Coatings: The Interplay of the Size and Loading of Particles

Nusret Celik[1,2], *Furkan Sahin*[1], *Sultan Suleyman Ozel*[2], *Gulay Sezer*[3], *Nail Gunaltay*[1], *Mahmut Ruzi*[1,*], *M. Serdar Onses*[1,2,4,*]

[1] ERNAM - Erciyes University Nanotechnology Application and Research Center, Kayseri, 38039, Turkey

[2] Department of Materials Science and Engineering, Erciyes University, Kayseri, 38039, Turkey

[3] Department of Pharmacology, Erciyes University, Faculty of Medicine, 38039, Kayseri, Turkey

[4] UNAM−National Nanotechnology Research Center, Institute of Materials Science and Nanotechnology, Bilkent University, 06800, Ankara, Turkey

* Address correspondence to: onses@erciyes.edu.tr, mruzi17@gmail.com

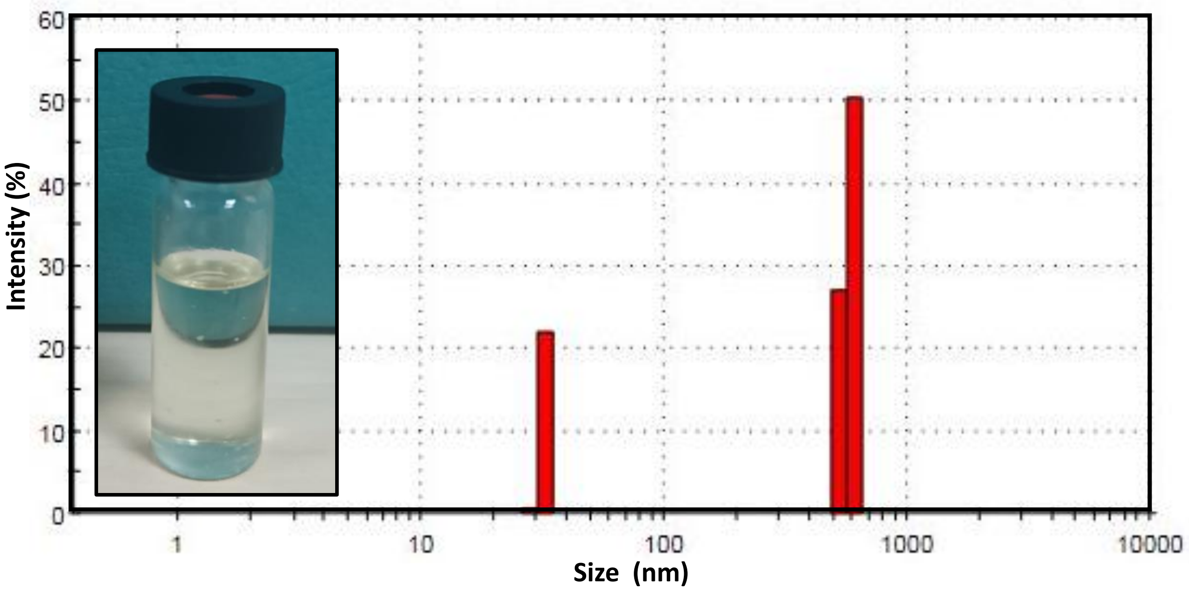

Figure S1. The size distribution of the dispersion used to prepare the superhydrophobic coating, which was measured via DLS (Nano ZS, Malvern). The average size is 461 nm. Shown in the inset is the photograph of the vial containing the dispersion used to prepare the superhydrophobic coating.

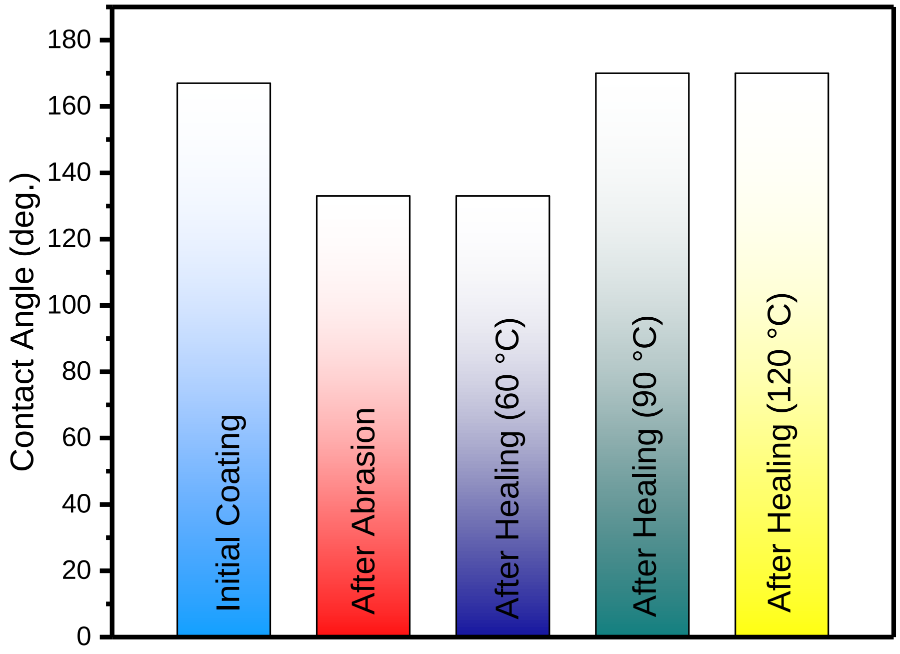

Figure S2. Effect of annealing temperature on the healing ability of the superhydrophobic coating.

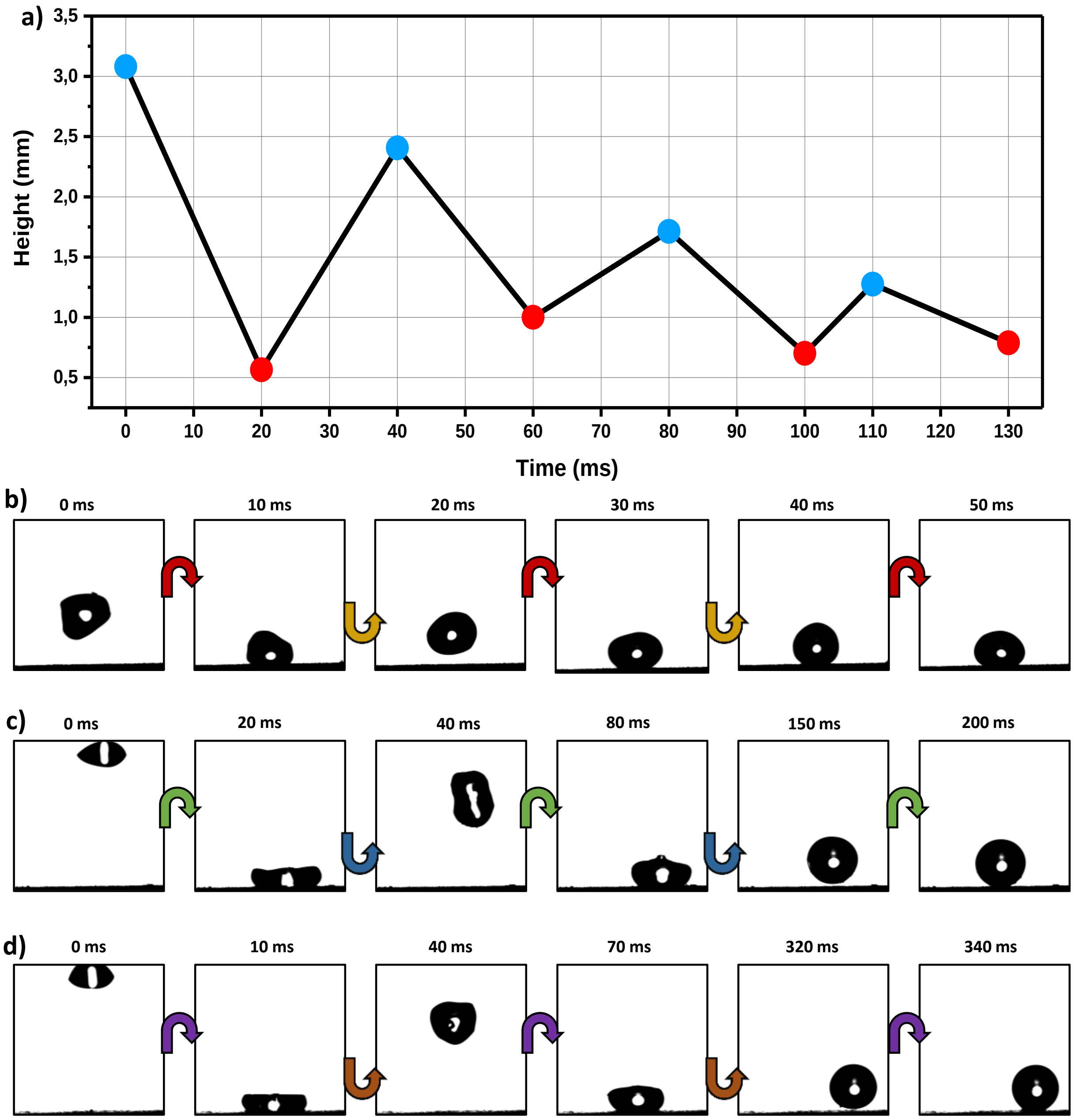

Figure S3. a) Temporal evolution of the center of gravity of a ~6 µL (R_0 = 1.2 mm) water droplet after release from 1 cm height, showing bouncing behavior on the initial superhydrophobic surface. b-d) Snapshots of the bouncing droplet on the b) initial coating (height: 1 cm), c) initial coating (height: 2 cm), and d) after healing (height: 2 cm).

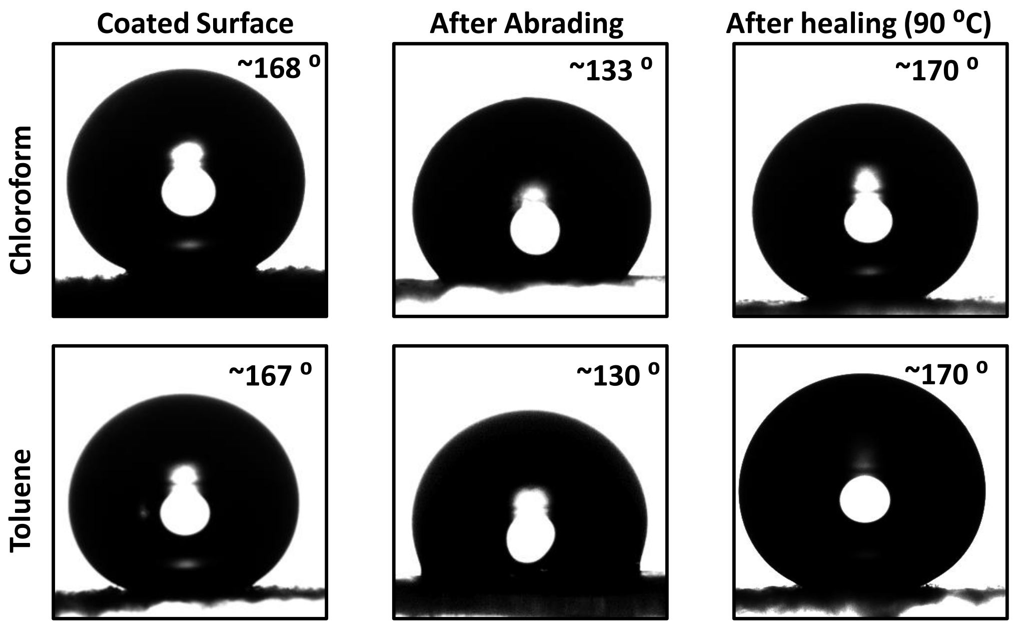

Figure S4. Water contact angle of the coatings prepared in different solvents, before and after abrasion, as well as after healing.

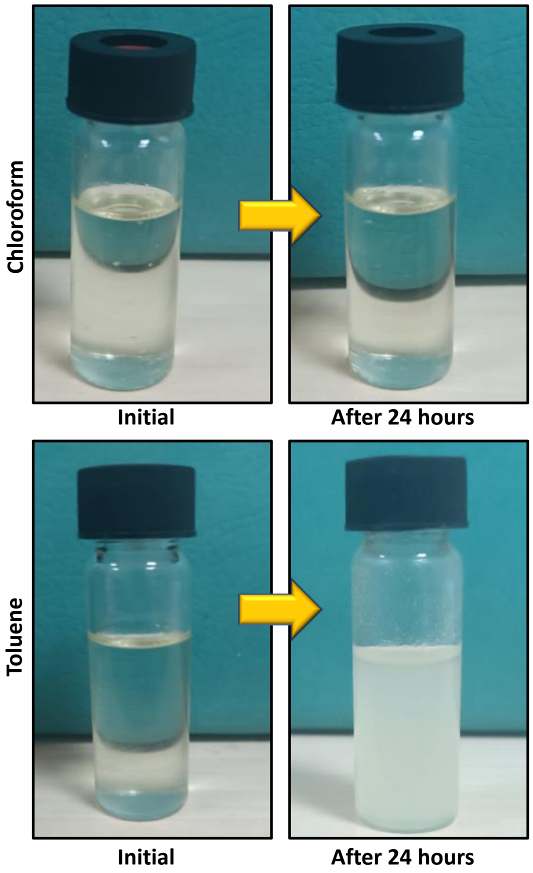

Figure S5. Photographs of the vials containing the initial dispersions and after being kept for 24 hours. The dispersion prepared in toluene is not stable and starts to become cloudy.

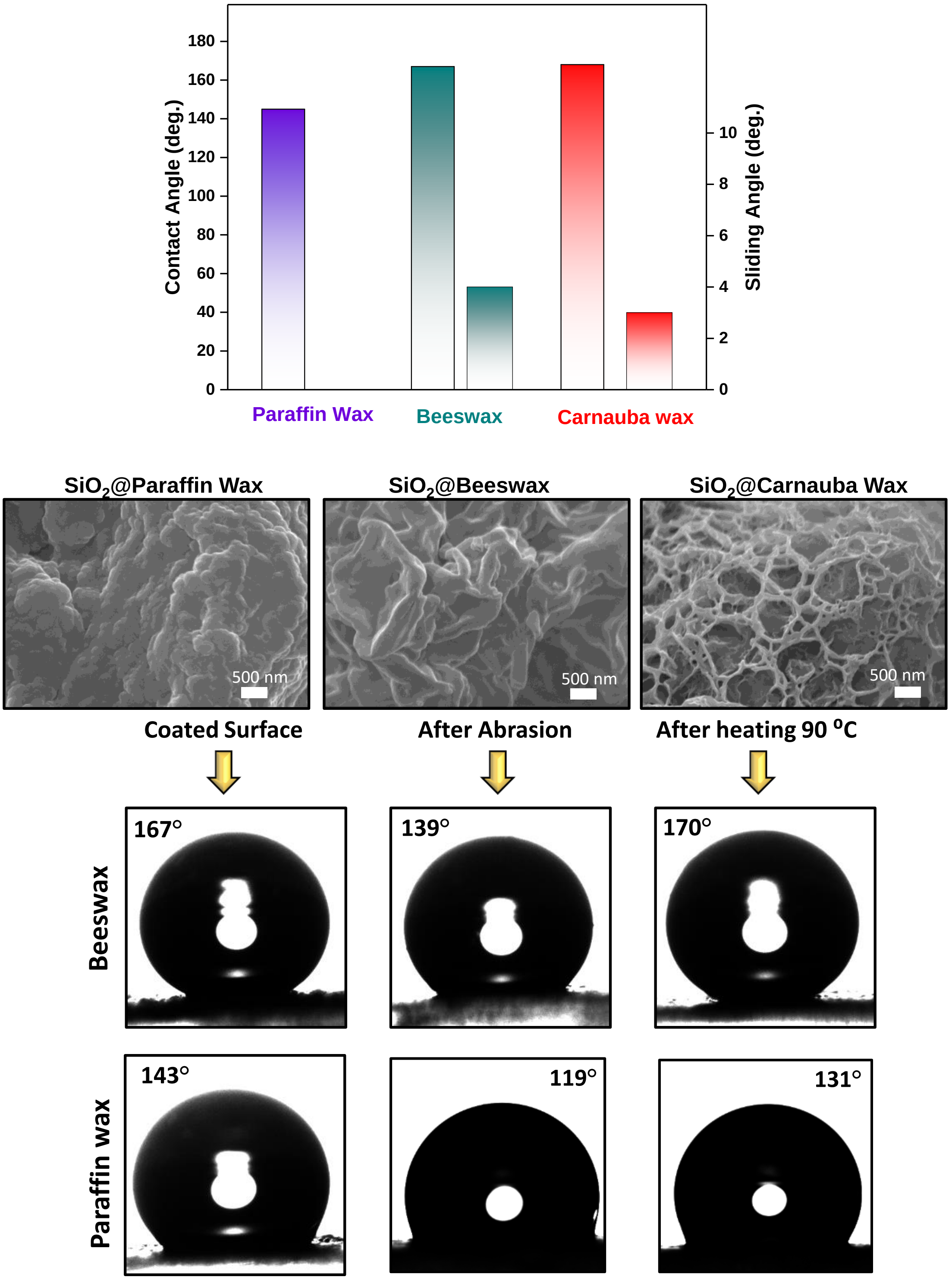

Figure S6. Wetting and healing properties of superhydrophobic coatings prepared from various waxes. Top: Water CA and SA values of healed surfaces prepared from various waxes. Middle: SEM images of the initial coatings. Also shown are the CA angle and pictures of a water droplet (10 μL) on the coatings prepared from beeswax and paraffin wax.

Table S1. Chemical characterization of the coatings using XPS.

Atomic (%)	C	O	Si
Initial coating	83.61	11.74	4.65
After abrasion	53.21	29.72	17.06
After heating	63.22	22.05	14.73

Table S2. Water SA of the coatings prepared from silica nanoparticles of different sizes before and after abrasion, as well as after healing.

Size of particles	Initial Coating SA (°)	After Abrading SA (°)	After healing SA (°)
11 nm	4	No droplet sliding observed	2
90 nm	4	No droplet sliding observed	4
260 nm	3	No droplet sliding observed	4
4 µm	2	No droplet sliding observed	No droplet sliding observed
8 µm	2	No droplet sliding observed	No droplet sliding observed
20 µm	2	No droplet sliding observed	No droplet sliding observed

Table S3. Change of water SA of the coating as a function of abrasion/healing and tape peeling/healing cycles. The SA of the initial coating is 4°.

**Number of Cycles**	**After Abrading SA (°)**	**After Healing SA (°)**	**After Tape peeling SA (°)**	**After Healing SA (°)**
1	No droplet sliding observed	2	No droplet sliding observed	4
2	8	2	12	4
3	12	5	12	5
4	10	7	15	6
5	14	8	15	9

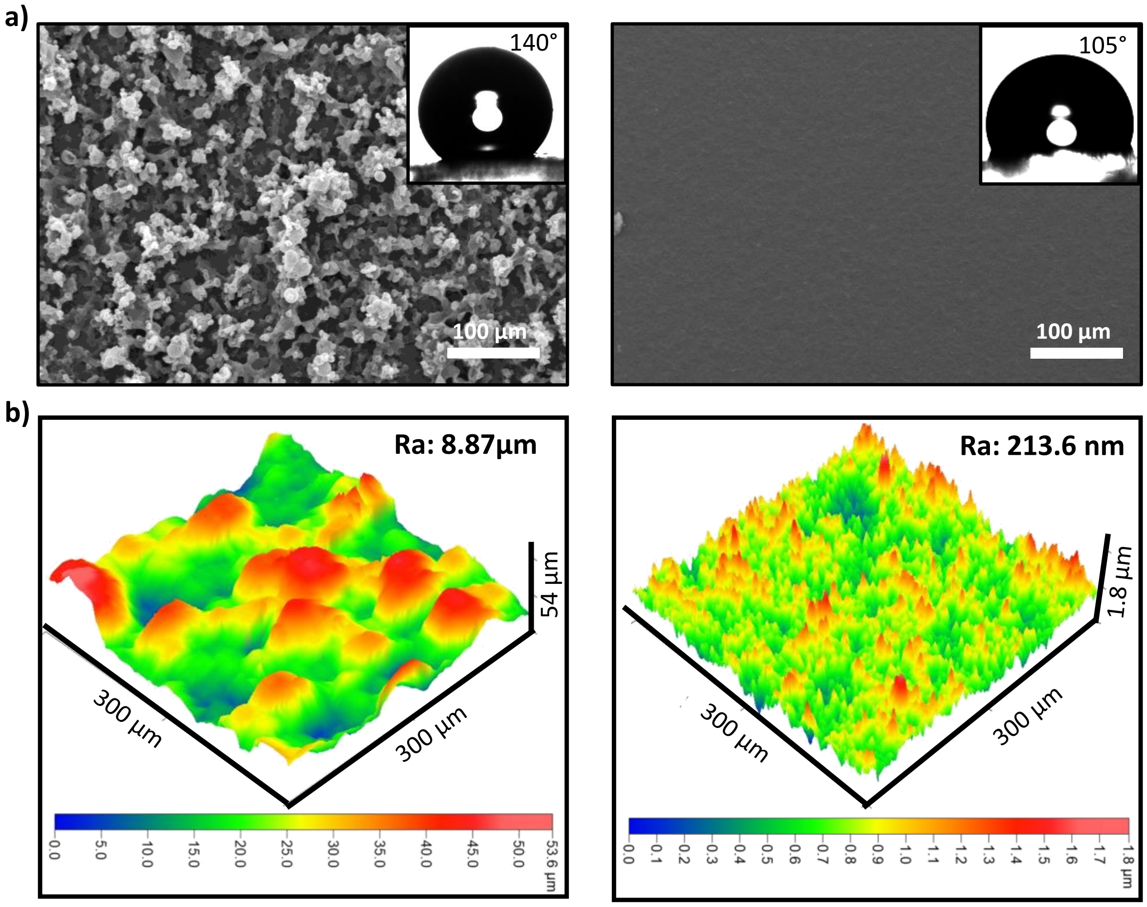

Figure S7. Characterization of the wetting and surface topography of the coating prepared by only using carnauba wax. a) SEM images of the initial coated surface (left) and after heating (right). The inset shows water CA. b) Surface topography of the corresponding surfaces.

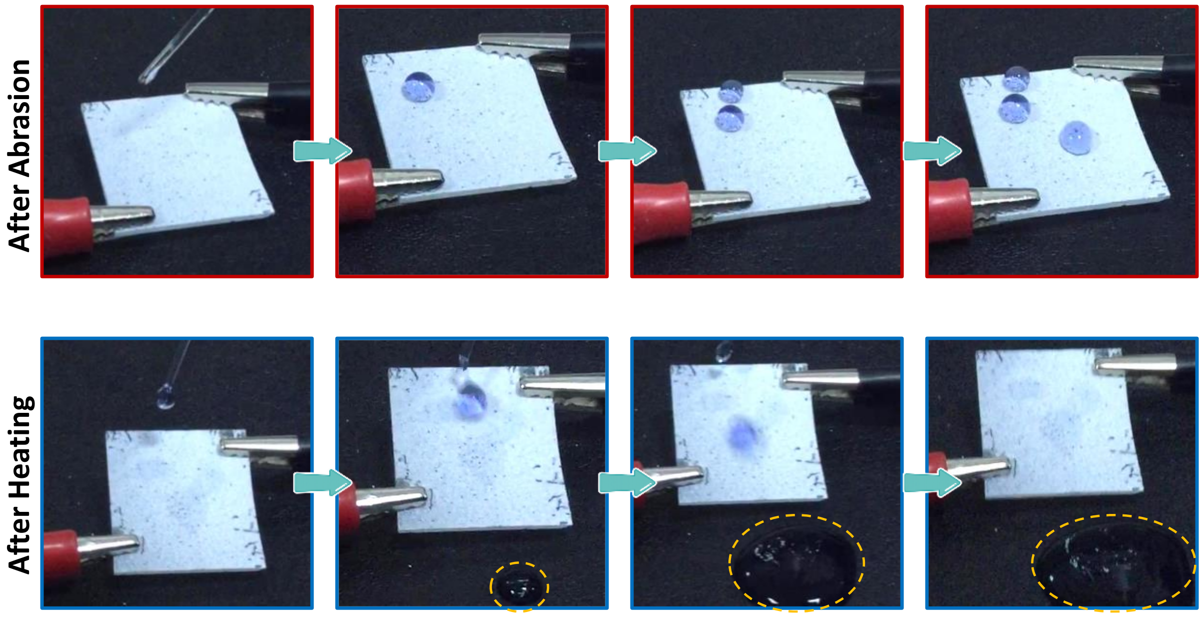

Figure S8. Demonstration of self-healing ability of the superhydrophobic coating on an ITO substrate achieved by electrical heating.

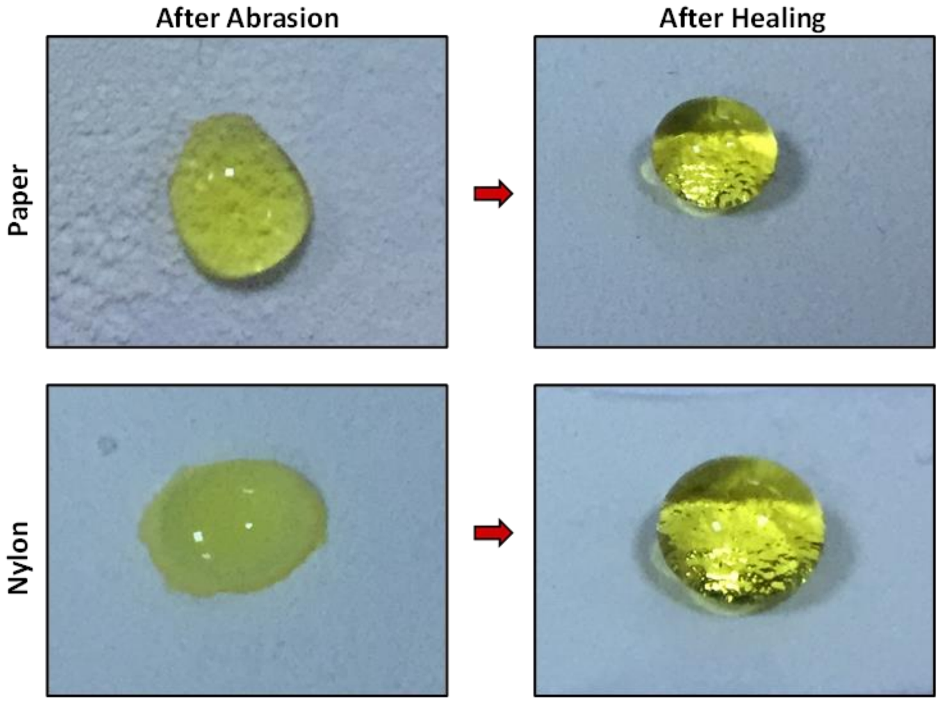

Figure S9. Demonstration of the self-healing on different substrates.

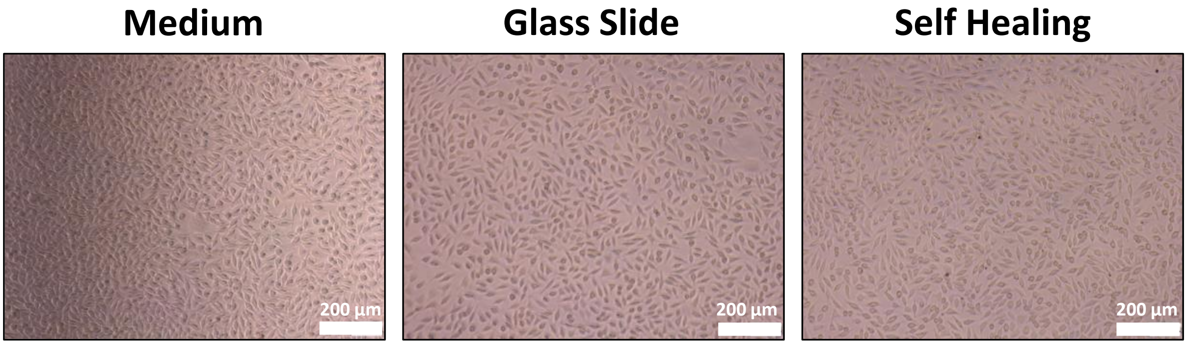

Figure S10. Cell morphology of L929 cells when cultured with medium, glass slide, and self-healing substrate medium extracts, respectively, for 24 h.
